# Reply to Khader, Y.S. Comment on “Alkouri et al. Metabolic Syndrome in Middle Eastern Patients with Atherosclerotic Cardiovascular Disease: A High Burden Driven by Cumulative Risk Factors. *J. Cardiovasc. Dev. Dis.* 2026, *13*, 240”

**DOI:** 10.3390/jcdd13090454

**Published:** 2026-09-10

**Authors:** Osama Alkouri, Walid Al-Qerem, Mohamad Jarrah, Ghaleb Alharbi, Nour Ali Alrida, Rahma Musaed Alabkal, Ayman Jaber Hammoudeh, Mohamed Ezzelregal Abdelgawad, Abdulkareem Alshehri, Abdullah Yaqoub Hasan, Mohannad AbuRuz, Fatma Refaat Ahmed, Mohammed Aldalaykeh

**Affiliations:** 1 Faculty of Nursing, Yarmouk University, P.O. Box 566, Irbid 21163, Jordan; 2 Faculty of Pharmacy, Al-Zaytoonah University of Jordan, Amman 11733, Jordan; 3 Department of Internal Medicine, Faculty of Medicine, Jordan University of Science and Technology, Irbid 21110, Jordan; 4 Department of Clinical Pharmacy, College of Pharmacy, Shaqra University, Shaqra 11961, Saudi Arabia; 5 Department of Pharmacy, Ministry of Health, Kuwait City 13001, Kuwait; ralabkal@hotmail.com; 6 Department of Cardiology, Istishari Hospital, Amman 11184, Jordan; 7 Medical Surgical Nursing Department, College of Nursing, Jouf University, Sakaka 72388, Saudi Arabia; 8 Advanced Diagnostic and Therapeutic Institute, King Abdulaziz City for Science and Technology (KACST), Riyadh 12354, Saudi Arabia; 9 College of Nursing, The Public Authority for Applied Education and Training, Kuwait City 72853, Kuwait; 10 College of Health Sciences, University of Sharjah, Sharjah 27272, United Arab Emirates; 11 College of Nursing, QU Health Sector, Qatar University, Doha P.O. Box 2713, Qatar

## 1. Response to the Commentary

We sincerely thank Professor Yousef Khader for his careful reading of our article [1] and for his thoughtful and constructive comments [2]. We greatly appreciate his recognition of the importance of investigating metabolic syndrome (MS) among Middle Eastern patients with established atherosclerotic cardiovascular disease (ASCVD). We welcome the opportunity to clarify several methodological aspects of our study and to further place our findings within their appropriate scientific context. 

The commentator’s first concern relates to the overlap between standard modifiable risk factors (SMuRFs), the diagnostic components of metabolic syndrome, and the variables included in the regression analysis. We fully acknowledge that hypertension and lipid-related abnormalities are shared components of both SMuRFs and MS. However, this reflects the biological architecture of cardiometabolic disease rather than a methodological oversight. Importantly, our study did not seek to establish a causal relationship between SMuRF burden and metabolic syndrome. Rather, its primary objective was to describe the burden of MS across clinically relevant SMuRF categories in patients with established ASCVD and to characterize the clinical profile associated with the syndrome.

Furthermore, this issue was explicitly acknowledged in the published manuscript, where we stated that “because hypertension, dyslipidemia, diabetes mellitus, BMI, triglycerides, and HDL-C were included in both the exposure and outcome definitions, the observed increase in MS prevalence with the number of SMuRFs was partly expected,” and that the identified predictors should therefore be interpreted cautiously because of their overlap with the defining criteria of MS. Consequently, we believe that the reported associations should be interpreted as descriptive and clinically informative rather than as evidence of causality. 

The commentator also questions our operational definition of metabolic syndrome. As clearly described in the Methods Section, our definition was intentionally adapted because waist circumference and fasting glucose were not consistently available across all contributing cardiovascular registries. Rather than excluding a substantial number of otherwise eligible participants or limiting the analysis to a single registry, we adopted a harmonized definition using variables that were uniformly available across all datasets. This approach is common in pooled registry analyses, where harmonization of available variables is often necessary to maximize data completeness and preserve statistical power.

We explicitly acknowledged this methodological consideration in both the Methods and Limitations Sections and cautioned readers regarding interpretation. Therefore, while direct numerical comparisons with studies using the complete NCEP ATP III criteria should be undertaken cautiously, we do not believe that the modified definition invalidates comparisons with the broader literature or diminishes the internal validity of the observed associations within our study population.

Regarding the pooled analytical cohort, the commentator expresses concern about combining one prospective cohort with multiple retrospective registries and about the reduction from the overall registry populations to the final analytical sample. Individual participant data pooling from multiple complementary cohorts is a well-established epidemiological approach that increases statistical precision, broadens clinical representation, and allows investigation of relatively uncommon patient subgroups that may not be adequately represented within individual studies.

Before pooling, variables from the contributing registries were harmonized post hoc to common operational definitions, and uniform eligibility criteria were applied across all participating datasets. The reduction from the combined registry populations to the final analytical cohort resulted from predefined eligibility criteria together with the requirement for complete information on all variables necessary to define metabolic syndrome and perform the planned analyses. The original registries were established to address different cardiovascular research questions and therefore included many participants who were outside the scope of the present investigation or lacked complete data for the variables required for this specific analysis.

These considerations were also acknowledged in the published Limitations Section, where we noted the potential for methodological heterogeneity, selection bias arising from complete-case analysis, and limited generalizability inherent in pooled registry studies. Accordingly, our conclusions were intentionally confined to the studied population of patients with established ASCVD rather than implying unrestricted generalization to all Middle Eastern populations.

Finally, with respect to the statistical analyses, all analyses were conducted according to a prespecified statistical plan appropriate for the observational design and available data. As discussed in the manuscript, the observational nature of the study precludes causal inference, and residual confounding cannot be excluded. These limitations were explicitly acknowledged. Nevertheless, the principal observation of a progressive increase in metabolic syndrome prevalence with increasing cardiometabolic risk burden remained robust and biologically plausible. Similarly, the identified clinical correlates—including older age, diabetes mellitus, hypertension, chronic kidney disease, heart failure, elevated BMI, elevated triglycerides, and lower HDL-C—are consistent with the established pathophysiology of metabolic syndrome and with the previously published literature.

## 2. Conclusions

In conclusion, we sincerely appreciate Professor Khader’s thoughtful observations and his valuable engagement with our work. Most of the issues raised—including the modified operational definition of metabolic syndrome, the overlap between SMuRFs and MS components, the observational nature of the analyses, and the methodological considerations inherent in pooled registry data—were explicitly recognized and discussed in the published manuscript. We therefore respectfully submit that these limitations should be interpreted within the context of the study design and should not detract from the principal contribution of the study, namely, demonstrating the substantial burden and clustering of metabolic syndrome among Middle Eastern patients with established ASCVD using one of the largest harmonized cohorts currently available from the region.

## Data Availability

Data Are available upon reasonable request from the corresponding author.
